# Three Outbreaks of COVID-19 in a Single Nursing Home over Two Years of the SARS-CoV-2 Pandemic

**DOI:** 10.14336/AD.2022.0624

**Published:** 2023-02-01

**Authors:** Vladan Čokić, Zorana Popovska, Olivera Lijeskić, Ljiljana Šabić, Olgica Djurković-Djaković

**Affiliations:** ^1^Institute for Medical Research, University of Belgrade, Belgrade, Serbia; ^2^Nursing Home Lug, Mladenovac, Serbia

**Keywords:** COVID-19, SARS CoV-2 variants, nursing home, residents, staff, outcome, case fatality rate, sex-related differences, SARS-CoV-2 specific antibodies

## Abstract

Older people in nursing homes (NH) have been hit particularly hard by the COVID-19 pandemic. We conducted a retrospective study of three outbreaks of COVID-19, occurring during the waves of the initial pre-Alpha, Delta and Omicron SARS-CoV-2 variants, in one NH in suburban Belgrade, Serbia. All staff and 95% residents were vaccinated in February 2021, mostly with BBIBP-CorV, and two thirds were boosted with a third dose in August 2021. COVID-19 was diagnosed by positive PCR and/or antigen test. After the first outbreak, 80 affected individuals were tested for SARS-CoV-2 specific antibodies. The first outbreak involved 64/126 (50.8%) residents and 45/64 (70.3%) staff, the second 22/75 (29.3%) residents and 3/40 (7.5%) staff, and the third involved 36/110 (32.7%) residents and 19/56 (33.9%) staff. Clinical presentation ranged from asymptomatic to severe, with severe cases referred to hospital ICUs. Deaths occurred only in residents, and the case fatality rate was 31.2%, 9.1% and 0%, respectively in outbreaks 1, 2 and 3. Specific IgG antibodies were detected in all 35 residents and 44 of the 45 staff, and higher IgG levels were detected in the residents (417.3±273.5) than in the staff (201.9±192.9, p<0.0001) despite a double difference in age (79.0±7.4 vs. 40.1±11.5 years). Outbreaks 2 and 3 involved four and 23 breakthrough infections, respectively. Older individuals mounted a good immunological response to SARS-CoV-2 infection and vaccination, which prevented significant mortality and severe morbidity in the subsequent outbreaks, despite a significant number of breakthrough infections.

The coronavirus disease 2019 (COVID-19) pandemic has been particularly devastating for nursing home (NH) residents, as older people are at a higher risk of severe disease and death and living in close contact favors transmission which can hardly be avoided at times of high community incidence [1, 2]. This has been particularly true in the first year of the pandemic, before the availability of vaccines. Several studies carried out in Europe and the US have shown that early into the pandemic, outbreaks occurred in more than a half of long-term care facilities (LTCFs) [3], and the reported incidences ranged from 22% to 47% in residents and from 24% to 45% in staff, while the case fatality rates (CFR) ranged between 27% and 36.9% in residents [1, 4-9].

The disease severity and CFR in older people compared to other age groups has been associated with co-morbidities as a major risk factor for poor prognosis [10]. Older age and male sex were shown to correlate with the severity of SARS-CoV-2 infection, but interestingly, sex differences became less important with advancing age [11,12]. In addition, LTCFs experienced 34.8% excess deaths, without probable or confirmed Covid-19 diagnoses, compared to historical estimates [13].

Due to the above, LTCFs were a priority target for vaccination when it started in early 2021. Vaccination did in fact bring considerable relief by significantly reducing transmission and infection and changed the above patterns in older adults. A well-known Israeli study based on national surveillance data after a nationwide vaccination campaign found that the mRNA BNT162b2 (Pfizer-BioNTech) vaccine maintained high effectiveness in the general population of older adults, including adults aged 85 or older [14]. However, outbreaks of COVID-19, including severe cases and deaths, were still shown to occur in NHs despite full vaccination of most residents [15]. Failures are associated with the constant load of new mutations. Reports from 3,862, 11,581 and 14,917 NHs from the pre-Delta period, intermediate period (May-June 2021) and the Delta period demonstrated adjusted effectiveness against SARS-CoV-2 infection of 74.7%, 67.5% and 53.1%, respectively for any mRNA vaccine [16].

Here we report on three COVID-19 outbreaks in one NH in the suburbs of Belgrade, Serbia, which occurred during the waves of the initial pre-Alpha (Nov 2020), Delta (Nov 2021) and Omicron variants (Jan 2022) of SARS-CoV-2, respectively. The objective of the study was to describe and analyze characteristics of infection, transmission, and outcome in parallel among the residents and staff in each outbreak, with particular emphasis on sex-related differences. Moreover, since the first outbreak occurred pre-vaccination and the other two after the vaccination campaign, we compared the characteristics of the outbreaks before and after vaccination and analyzed vaccine effectiveness.

## MATERIAL AND METHODS

### Study design, setting and study population

We conducted a retrospective observational study of the epidemiological and clinical characteristics of COVID-19 during three different waves of SARS-CoV-2 infection in one licensed private NH located in a suburb of Belgrade. The NH consists of two buildings, with a total capacity of 160 residents (64 in object A and 96 in object B). At the time of the outbreaks, monthly occupancy ranged between 60% and 72%. The residents were serviced by a staff of 56 to 64, which included one medical doctor (general practitioner) and 13 nurses.

Medical records of all affected individuals were reviewed and analyzed for age, sex, group category (i.e., staff or resident), hematological test results, co-morbidities, immunization status, therapy, infection-related symptoms, hospitalization, and clinical outcome. In addition, we analyzed the number of deaths during the pandemic compared with the preceding years.

The study followed the principles of the Declaration of Helsinki. Participants gave informed consent for participation in the serological testing; the protocol has been approved by a local (Institute for Medical Research) Ethics Committee (approval no. EO138/20 and no. EO138/21).

### Detection of SARS-CoV-2

The start of the COVID-19 outbreak in the NH was defined as the date when the first symptoms appeared in the first resident and were confirmed by a SARS-CoV-2 PCR-positive nasopharyngeal swab. The COVID-19 testing campaign was implemented between November 2020 and February 2022, during epidemic outbreaks in the NH by the Belgrade City Institute for Public Health. All swabs were collected in-house, while the testing itself was carried out at official regional PCR laboratories, including the Belgrade City Institute of Public Health and the Institute of Virology, Vaccines and Sera "Torlak", Belgrade. Prior to PCR testing, any suspicion of COVID-19 symptoms or contacts in both residents and staff was checked by antigen SARS-CoV-2 testing (CHIL COVID-19 Antigen Rapid Test, Chil Tıbbi Malzeme Sanayi ve. Ticaret Limited Şirketi, Cigli-Izmir, Turkey) at the NH itself. This combined antigen and PCR testing served as a protective epidemiological measure instigated by any COVID-19 related symptom.

### Epidemiological measures and medical monitoring strategy

The NH applied epidemiological measures recommended for NHs by the Serbian Ministry of Labor, Employment, Veteran and Social Affairs. Staff working in COVID-19 areas used personal protective equipment consisting of gowns, gloves, face shields, and respirator masks. SARS-CoV-2-positive residents were isolated in dedicated areas and received standardized on-site care. The on-site physician, supported by an infectious disease specialist from the local community hospital, oversaw all treatments, and determined patients requiring intensive care, who were referred to Belgrade hospital ICUs. Strategies used to prevent and control the transmission of COVID-19 included social distancing and isolation of residents and staff, zoning and cohorting of residents with restriction of movement and activities, ban on visits and specific staff working arrangements.

### Testing for SARS-CoV-2 specific antibodies

A subset of individuals affected during the first outbreak was tested for SARS-CoV-2 specific antibodies, at 4-8 weeks and 9-12 weeks following the outbreak. Blood samples were collected at both time points into serum clot activator tubes (Vacuette CAT Serum Clot Activator, Greiner Bio-One, Kremsmünster, Austria) at the NH and transported without delay to the Institute for Medical Research for further analysis. Upon receival, tubes were centrifuged for 17 minutes at 2200 x g, and sera were analyzed immediately. After analysis, all serum samples were permanently stored at -80 °C. Detection of IgM and IgG antibodies specific for the receptor-binding domain (RBD) of the SARS-CoV-2 spike protein was performed on a MINI VIDAS analyzer using VIDAS SARS-CoV-2 IgM and VIDAS SARS-CoV-2 IgG II kits (BioMérieux, Marcy l’Etoile, France). Both assays are based on an enzyme linked fluorescence technique (ELFA), a two-step sandwich ELISA method combined with final fluorescence detection. The results were expressed as indices, and all values higher than or equal to 1 were considered positive, as per the manufacturer’s recommendation. Indices for IgG antibodies were converted to Binding Antibody Units/ml (BAU/ml), according to a conversion factor of 20.33 provided by the manufacturer.

### Statistical analysis

Normality of the distribution of the data was examined by the Shapiro-Wilk and the Kolmogorov-Smirnov tests. When the distribution was not normal, Mann-Whitney was used for intergroup comparisons, and Kruskal-Wallis with Dunnett’s post hoc test for multiple comparisons. Differences in the distribution of parametric data were tested by one-way ANOVA and t-test, as appropriate, followed by Dunn’s or Tukey’s post-hoc tests for multiple comparisons, as well as Pearson’s correlation. All statistics were performed using Prism 6 software (GraphPad Software Inc., San Diego, CA, USA). The results are expressed as the mean ± SD or SEM, as appropriate. The level of significance was 5%.

## RESULTS

### Epidemiological overview

From the beginning of the pandemic up to February 2022, a total of three outbreaks of COVID-19 occurred in the observed NH, involving a total of 189 infections, of which 122 (64.6%) in residents and 67 (35.4%) in the staff. The basic characteristics of all patients involved in all outbreaks are presented in Table 1. Most cases of infection occurred in outbreak 1 (109, 57.7%), which interestingly, involved proportionally more of the current total staff (70.3%) than of the total residents (50.8%). In contrast, outbreak 2 involved the lowest number of patients (13.2%), affecting 29.3% of all residents but only 7.5% of the staff, while outbreak 3 involved 55 cases (29.1%), evenly distributed between the residents (32.7%) and the staff (33.9%). The mean resident age did not differ among the outbreaks, but was exactly double (80.9 ± 9 years) (Mann-Whitney test, p<0.0001) that of the staff (41.04 ± 11.96 years) in all outbreaks.

**Table 1:** Characteristics of individuals involved in three outbreaks of COVID-19 in a single nursing home.

	Outbreak 1 Nov 2020		Outbreak 2 Nov 2021		Outbreak 3 Jan 2022	
	Residents (n)	Staff (n)	Residents (n)	Staff (n)	Residents (n)	Staff (n)
Total ill	64/126 (50.8%)	45/64 (70.3%)	22/75 (29.3%)	3/40 (7.5%)	36/110 (32.7%)	19/56 (33.9%)
Sex						
Female	40 (62.5%)	35 (77.8%)	15 (68.2%)	2 (66.7%)	19 (52.8%)	17 (89.5%)
Male	24 (37.5%)	10 (12.2%)	7 (31.8%)	1 (33.3%)	17 (47.2%)	2 (10.5%)
Age (mean ± SD)	80.95 ± 8.69	41.11 ± 11.52	79.23 ± 11.51	45.67 ± 18.77	81.92 ± 7.86	40.16 ± 12.52
Severity						
Asymptomatic	8 (12.5%)	16 (35.5%)	2 (9.1%)	1 (33.3%)	1 (2.8%)	4 (21.1%)
Mild	25 (39.1%)	25 (55.5%)	13 (59.1%)	2 (66.7%)	29 (80.5%)	16 (63.1%)
Moderate	9 (14.1%)	4 (8.9%)	3 (13.6%)	0 (0%)	4 (11.1%)	3 (15.8%)
Severe	22 (34.4%)	0 (0%)	4 (18.2%)	0 (0%)	2 (5.6%)	0 (0%)
Case fatality rate	20/64 (31.2%)	0 (0%)	2/22 (9.1%)	0 (0%)	0 (0%)	0 (0%)
Vaccine						
Two doses (Feb 2021)	/	/	22 (100%)	3 (100%)	33 (91.7%)	19 (100%)
Third dose (Sep 2021)	/	/	8 (36.3%)	2 (66.7%)	22 (61.1%)	13 (68.4%)

**Figure 1.**
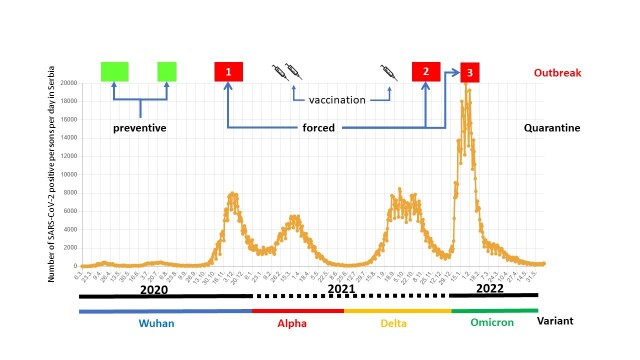
Timeline of three COVID-19 outbreaks in the nursing home (NH). Outbreaks in the NH (red boxes with ordinal number of outbreak) according to the duration of quarantine (preventive - green, forced - red), vaccination schedule, SARS-CoV-2 variants, and the number of SARS-CoV-2 positive persons per day in Serbia (from March 2020 through May 2022) as reported by the Institute of Public Health of Serbia "Dr Milan Jovanović Batut" (https://covid19.data.gov.rs).

**Figure 2.**
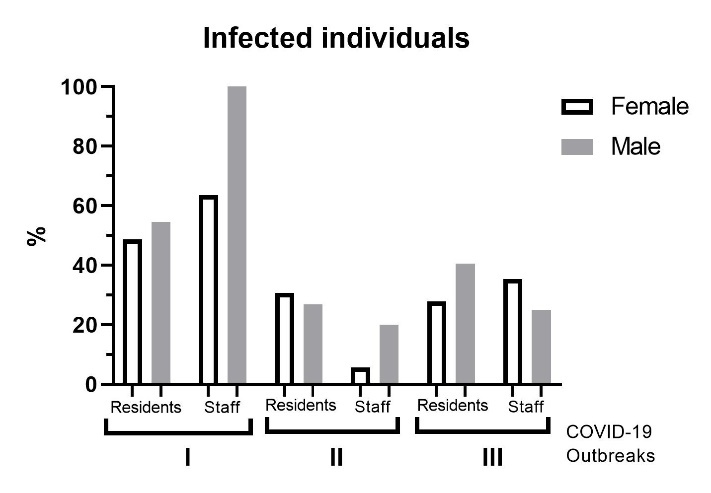
Sex-related morbidity in the nursing home (NH) during the COVID-19 pandemic. The male to female ratios were calculated in relation to the total number of residents and staff at the time of each outbreak of COVID-19 in the NH. The number of infected residents according to sex was 40/82 (48.8%) females (F) and 24/44 (54.5%) males (M) in the first outbreak, 15/49 (30.6%) F and 7/26 (26.9%) M in the second and 19/68 (27.9%) F and 17/42 (40.5%) M in the third outbreak. The number of infected staff was 35/54 (63.6%) F and 10/10 (100%) M in the first outbreak, 2/35 (5.7%) F and 1/5 (20%) M in the second and 17/48 (35.4%) F and 2/8 (25%) M in the third outbreak.

The timeline of all three outbreaks is presented in Fig. 1. The first symptoms in the index patient, a staffer, appeared on 20 November 2020, with a great majority of infections developing over the next three weeks. However, sporadic cases (a total of five, of which two residents and three staffers) continued through mid-December. Importantly, the onset of the outbreak was immediately reported to the authorities, and forced quarantine was introduced the day of the appearance of the first case (20 Nov 2020), which lasted until 24 December 2020. Quarantine implied no entry in and out of the NH, either of the residents or the staff, and with replacement of the staff every 2 weeks. Replacement staff entry was conditioned on a negative PCR test for SARS-CoV-2 24h before entry. The second outbreak, in November 2021, was comparably not as grave, involving 22/75 (29.3%) residents and 3/40 (7.5%) staff. This one lasted from 4 November to 6 December 2021. The third outbreak, in January 2022, involved 36/110 (32.7%) residents and 19/56 (33.9%) staff, and lasted from 10 January to 13 February 2022 (Table 1). Like with the first outbreak, the appearance of the first case in the subsequent outbreaks led to immediate forced quarantine, but just for the residents given that by that time all staff was vaccinated. More than a half of those ill with COVID-19 both overall and in each outbreak were women (Table 1), although this difference was not significant (p>0.05). However, the proportion of affected males was higher notably in non-vaccinated staff (100% males vs. 63.6% females) in the first outbreak (Fig. 2).

### Clinical outcome

Clinical presentation in all three outbreaks ranged from asymptomatic to severe, according to WHO guidance for clinical management of COVID-19 (Living guidance for clinical management of COVID-19, WHO https://apps.who.int/iris/bitstream/handle/10665/349321/WHO-2019-nCoV-clinical-2021.2-eng.pdf). Mild and moderate cases were treated at the NH, while severe cases were referred to Belgrade hospital ICUs. Expectedly, most severe cases occurred during outbreak 1, involving more than a third (22/64, 34.4%) of all COVID-19 patients, while there were four and two severe cases in outbreak 2 and outbreak 3, respectively (Table 1). Most severe cases (22/28, 78.6%) ended fatally, particularly during the first, pre-vaccination outbreak (20/22, 86.3%). In outbreak 2 of the four severe cases, two ended fatally, while in outbreak 3 there were no fatal outcomes. Overall, deaths only occurred in residents, and the CFR was 31.2%, 9.1% and 0%, respectively for outbreak 1, 2 and 3 (Table 1).

The Table 2 presents other important clinical parameters of the course of disease including treatment, co-morbidities, and outcome. Arterial hypertension (HA), diabetes mellitus type II (DM II) and dementia were the major co-morbidities. Supportive oxygen therapy (up to 5 l/min) was largely used in the first two outbreaks. Progression of primary disorder was the major complication in clinical outcome.

Next, we analyzed the mortality rate of residents during the pandemic in comparison to the preceding years. Annual mortality rates did not vary significantly among the analyzed years (2019-2022, Fig. 3), although the annual mortality rate (calculated as an average of the number of deaths per number of residents in each particular month of a specific year) was increased in 2020 (5.4%), 2021 (4.6%) and 2022 (4.3%, up to May) compared to 2019 (3.5%). Moreover, the annual number of deaths in this period did not significantly vary in comparison with the five preceding years (2015 - 43, 2016 - 67, 2017 - 44, 2018 - 70, 2019 - 54, 2020 - 73, 2021 - 58 deaths). However, since the excess deaths in 2020 occurred only in the last trimester, when this one was compared with the last trimester in the preceding years, the number of deaths in residents in 2020 was significantly higher than in each preceding year: 2019 (p=0.0033), 2018 (p=0.0372), 2017 (p=0.0167), 2016 (p=0.0305), 2015 (p=0.0136, One-way ANOVA, Dunn's multiple comparison test).

**Table 2:** Therapy, co-morbidities and outcome in nursing home residents with COVID-19 per outbreak.

Therapy	Multivitamin			Antiviral		Antibiotic		Anticoagulant		Corticosteroid		Oxygen	
	Total	n	%	n	%	n	%	n	%	n	%	n	%
Outbreak 1	64	64	100	2	3.1	28	43.8	57	89.1	21	32.8	27	42.2
Outbreak 2	22	2	95.5	18	81.8	12	54.5	22	100	2	9.1	10	45.5
Outbreak 3	36	36	100	0	0	18	50	30	83.3	0	0	1	2.8
Co-morbidities		HTA		DM II		post CVA		Dementia /Alzheimer		CMP		BPH	
Outbreak 1	64	48	75	15	23.4	16	25	19	29.7	11	17.2	4	6.2
Outbreak 2	22	12	54.5	6	27.3	3	13.6	12	54.5	4	18.2	1	4.5
Outbreak 3	36	27	75	8	22.2	7	19.4	6	16.7	2	5.6	2	5.6
Outcome		No consequences		Lung fibrosis		Reduced mobility		Disease progression					
Outbreak 1	64	34	53.1	5	7.8			7	10.9				
Outbreak 2	22	16	72.7			4	18.2						
Outbreak 3	36	36	100										

HTA - arterial hypertension; DM II - diabetes mellitus type II; Post-CVA - post cerebrovascular accident; CMP - cardiomyopathy, BPH - Benign Prostatic Hyperplasia

### Hematological parameters

Key hematological parameters in the residents in all three outbreaks are presented in Table 3. Mean total leukocyte, neutrophil, and lymphocyte counts, as well as thrombocyte counts, remained within normal values in all outbreaks, and with no sex-related difference in outbreak 1 and 2. Conversely in outbreak 3, neutrophil and thrombocyte counts were higher (t-test, p=0.0003 and p=0.037, respectively), and lymphocyte counts lower (t-test, p<0.0001), in males than in females, although within the normal range of values. Neutrophil lymphocyte ratio (NLR) was also elevated in all outbreaks, with no sex-related differences in outbreak 1, while it was significantly higher (t-test, p=0.0003) in males in outbreak 3 (Table 3). Similarly, C reactive protein (CRP) values were elevated in both males and females in all three outbreaks, and significantly higher (t-test, p=0.001) in males in outbreak 3. D-dimer was analyzed only in the residents of outbreak 1, where a total of 44 patients (68.8%) had elevated D-dimer values. This elevation was light on average (range 0.2 - 358), and although apparently higher in females the difference in the mean level between the sexes was not significant (Mann-Whitney test, p>0.05) obviously due to large individual variations. However, when we analyzed blood cell counts according to clinical outcome, significant differences were steadily observed between cases without complications and cases with complications and lethal outcome (Fig. 4). Leukocyte (Kruskal-Wallis, Dunnett's multiple comparison test, p<0.05) and neutrophil (one-way ANOVA, Tukey's multiple comparison test, p<0.001) counts were significantly higher, while lymphocyte counts were lower (one-way ANOVA, Tukey's multiple comparison test, p<0.001), in cases with complications or that ended fatally (Fig. 4A-C). A similar pattern was registered for CRP and lactate dehydrogenase (LDH) (Fig. 4E, F). CRP levels were significantly increased in severe cases compared to mild (one-way ANOVA, Tukey's multiple comparison test, p=0.0002) and moderate (p=0.0036) cases in all three outbreaks of COVID-19. Taking all three outbreaks together, CRP levels were positively correlated with the severity of disease (Pearson’s r^2^=0.1177, p=0.0003).

**Table 3:** Main hematologic parameters in SARS-CoV-2-infected NH residents at the time of the diagnosis.

	Le	Ne	Ly	NLR	Tr	CRP	D-dimer	LDH
Normal value	4-10 x10^9^/L	42-76 %	17-48 %	1.7 ± 0.7	150-400 x10^9^/L	< 5 mg/L	< 0.5 ng/ml	208-378 U/L
Outbreak 1								
Total (mean ± SD)	6.7 ± 3.5	62.2 ± 14.2	27.3 ± 12.3	3.51 ± 3.7	233.5 ± 85.4	32.4 ± 42.9	9.6 ± 50.9	245.8 ± 142.9
Male	6.7 ± 3.1	61.7 ± 13.7	25.6 ± 10.9	3.4 ± 3.6	228.9 ± 86.2	44.9 ± 41.2	1.8 ± 1.3	290.7 ± 187.2
Female	6.8 ± 3.8	62.5 ± 14.9	28.3 ± 13.3	3.58 ± 3.8	235.5 ± 88.9	19.7 ± 40.9	14.9 ± 65.0	223.4 ± 120.0
P value	0.775	0.838	0.420	0.851	0.885	0.275	0.417	0.150
Outbreak 2								
Total (mean ± SD)	6.1 ± 1.9	61.9 ± 13.1	26.8 ± 12.9	3.01 ± 1.8	241.3 ± 84.6	17.7 ± 13.4		
Male	5.4 ± 0.5	66.7 ± 8.1	21.2 ± 6.6	3.48 ± 1.5	215.0 ± 57.6	18.9 ± 11.0		
Female	6.3 ± 2.1	60.3 ± 14.3	28.7 ± 14.1	2.85 ± 1.9	250.0 ± 94.3	17.2 ± 14.6		
P value	0.385	0.416	0.332	0.551	0.493	0.840		
Outbreak 3								
Total (mean ± SD)	5.7 ± 2.0	60.2 ± 9.9	27.6 ± 8.9	2.59 ± 1.5	210.8 ± 61.9	30.8 ± 31.8		
Male	6.1 ± 2.2	65.8 ± 8.3	22.2 ± 6.5	3.46 ± 1.7	186.4 ± 43.7	48.8 ± 36.7		
Female	5.2 ± 1.7	55.0 ± 8.2	32.8 ± 7.6	1.81 ± 0.7	230.9 ± 69.5	15.5 ± 15.7		
P value	0.140	0.0003	<0.0001	0.0003	0.037	0.001		

NLR - neutrophil lymphocyte ratio

**Figure 3.**
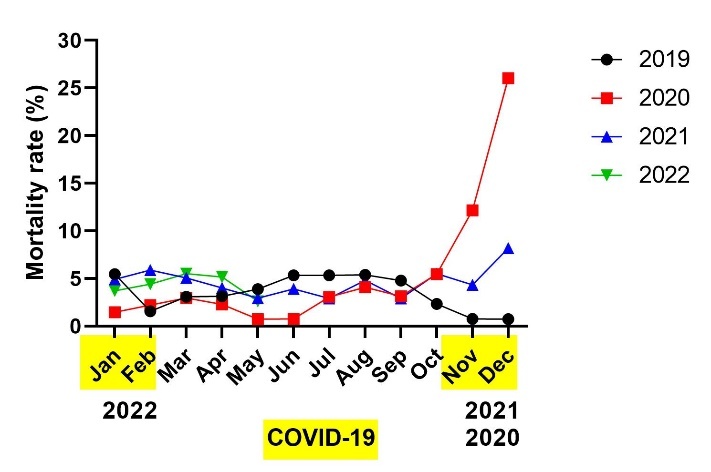
Mortality rate in the nursing home (NH) during the COVID-19 pandemic. The mortality rate is presented as the number of deaths per total number of NH residents per month. Months colored yellow correspond to forced quarantine, provoked by COVID-19, in the NH.

### Detection of specific IgG and IgM antibody

A subgroup of individuals affected in outbreak 1, consisting of both residents and staff, was tested for SARS-CoV-2 specific antibodies at two time points after the diagnosis of infection, the first one approx. 6 weeks for residents and 4.5 weeks for the staff, and the second one close to 6 weeks later for both categories. All 35 examined residents and 44 of the 45 examined staff were found to harbor specific IgG antibodies (Table 4). In the first sample, significantly higher levels were detected in the residents (417.3 ± 273.5) than in the staff (201.9 ± 192.9, Mann-Whitney, p<0.0001, Fig. 5A) despite a double difference in age (79.03 ± 7.43 years vs. 40.11 ± 11.52 years, Mann-Whitney, p<0.0001). At the follow-up time point there was waning of the specific humoral response in that specific IgG antibodies were no longer detectable in one resident (2.9%) and in five of the staff (13.9%), and the levels were lower than in the first sample (although not significantly; Mann-Whitney, p=0.18 and p=0.13, respectively for residents and staff). However, the IgG levels remained significantly higher in the residents (325.7 ± 214.4) than in the staff (137.7 ± 135.6, Mann-Whitney, p<0.0001, Table 4, Fig. 5A). As for the specific IgM antibodies, they were detected in 27/35 (77.7%) residents and in 23/45 (50%) staff and were borderline higher in residents (Mann-Whitney, p=0.052) (Table 4).

Analysis of the overall serological responses according to age showed no sex-related difference in specific IgG levels but interestingly, IgM indices were two-fold higher (Mann-Whitney, p=0.149) in males (5.92±8.06, n=22) than in females (2.87±4.14, n=58). However, analysis according to severity of clinical presentation (no symptoms (n=8), mild (n=17), moderate (n=6) and severe symptoms (n=4)) showed that residents with moderate symptoms had higher IgG levels (first sampling) than residents with mild (t-test, p=0.0001) and no symptoms (p=0.045), and borderline lower than residents with severe symptoms (p=0.0564, Fig. 5B). The findings in the follow-up sample were consistent with this, showing lower IgG levels in residents with mild symptoms than in those with moderate (t-test, p=0.001) and severe disease (p=0.0293, Fig. 5C). The IgM values in residents also tended to increase with the severity of clinical symptoms, albeit insignificantly (Mann-Whitney, p=0.1091, Fig. 5D).

**Figure 4.**
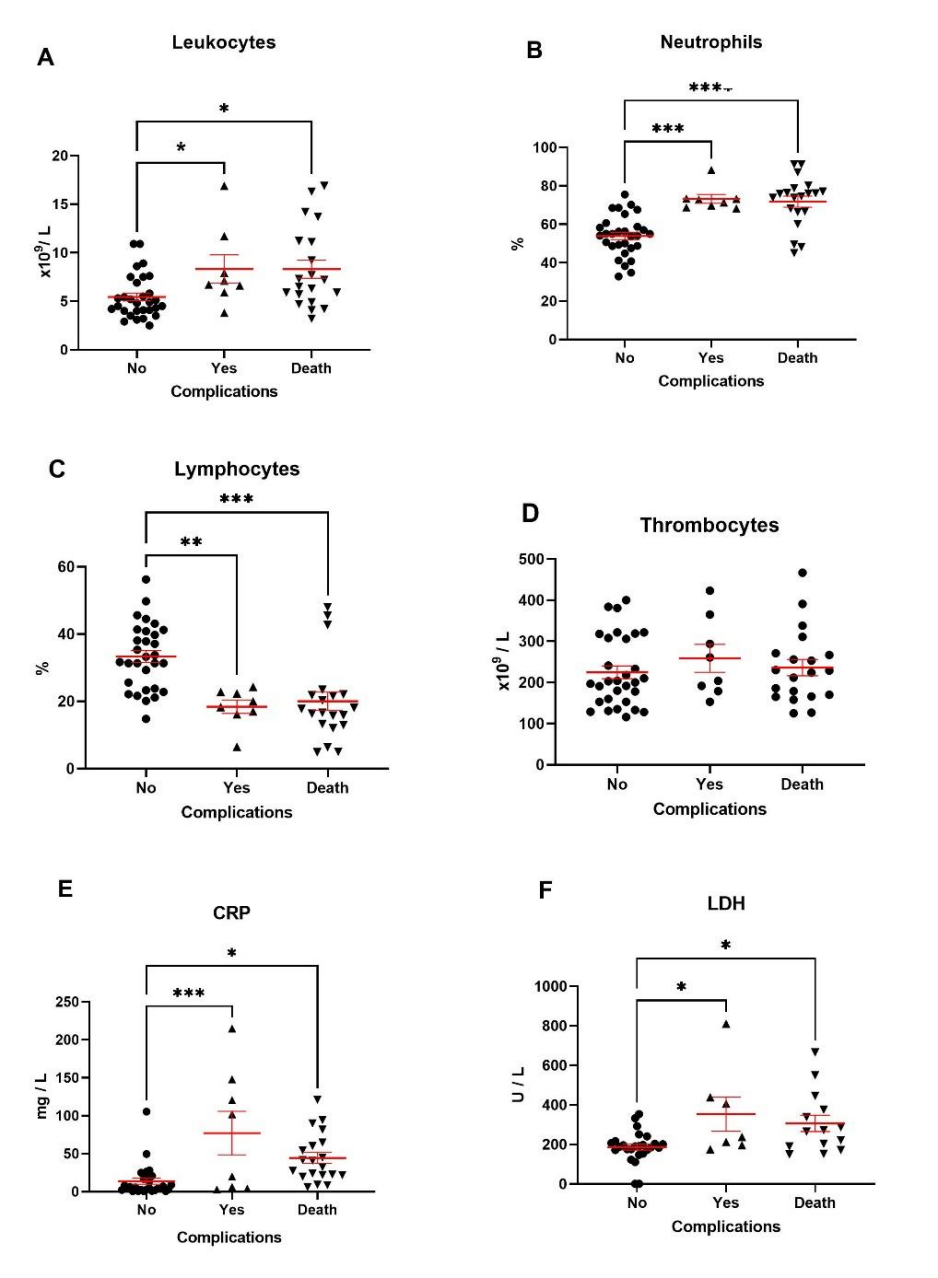
Main laboratory parameters at diagnosis of SARS-CoV-2 infection in residents during the first outbreak in the nursing home. (A) Leukocyte levels in patients with no post COVID complications (n=30), with post COVID complications (n=8) and in deceased patients (n=20). (B) Neutrophil levels in patients with no post COVID complications (n=30), with post COVID complications (n=8) and deceased patients (n=20). (C) Lymphocyte levels in patients with no post COVID complications (n=30), with post COVID complications (n=8) and in deceased patients (n=20). (D) Thrombocyte levels in patients with no post COVID complications (n=30), with post COVID complications (n=8) and in deceased patients (n=20). (E) CRP levels in patients with no post COVID complications (n=29), with post COVID complications (n=8) and in deceased patients (n=20). (F) LDH levels in patients with no post COVID complications (n=26), with post COVID complications (n=7) and in deceased patients (n=13). Values are expressed as mean ± SEM. *p<0.05, **p<0.01, ***p<0.001 vs. patients with no complications.

**Figure 5.**
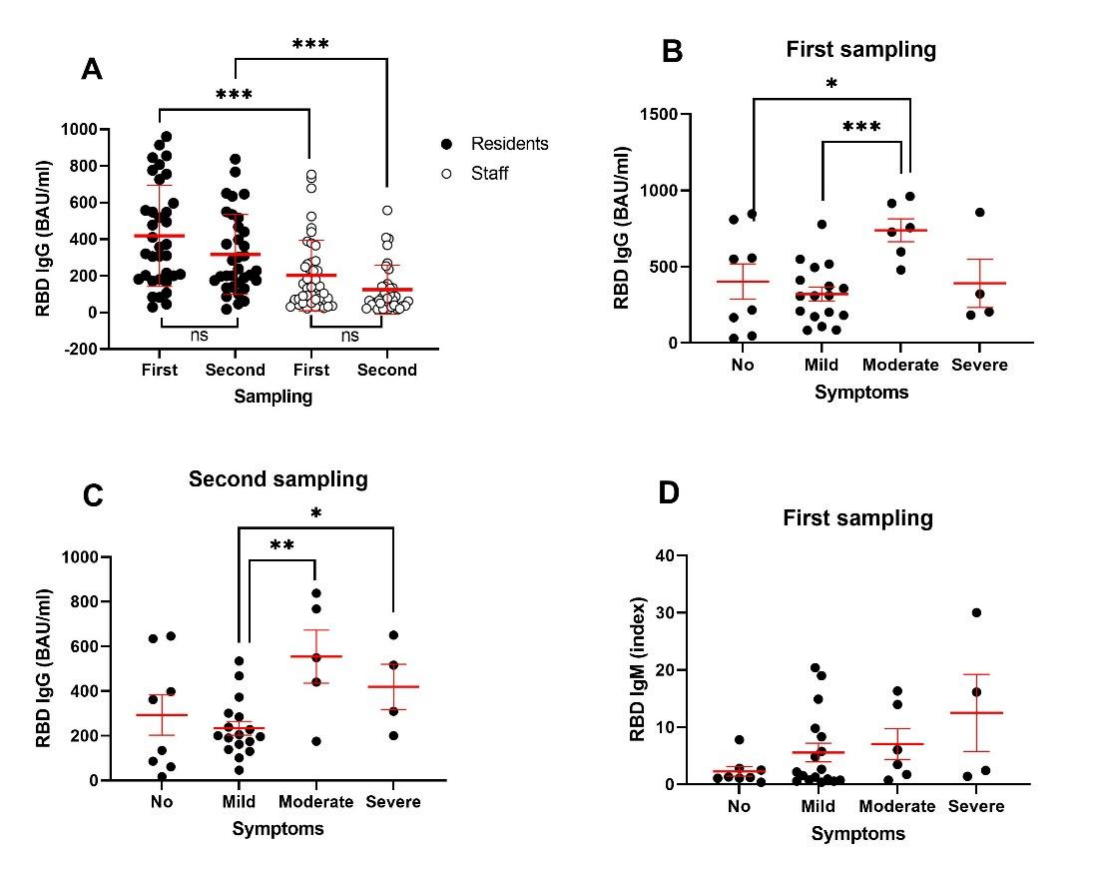
Levels of anti-RBD spike protein SARS-CoV-2 specific antibody in residents and staff after the first COVID-19 outbreak in the nursing home. (A) Specific IgG antibody at first sampling (after 40 and 30 days, respectively, in residents (n=35) and staff (n=44)) and second sampling (after 79 and 69 days, respectively, in residents (n=34) and staff (n=31)). Values are expressed as mean ± SD. Specific IgG antibody at (B) first sampling, and (C) second sampling, and (D) specific IgM antibody at first sampling, all according to severity of clinical presentation (no = asymptomatic). Values are expressed mean ± SEM. *p<0.05, **p<0.01, p<0.001.

### Active immunization and breakthrough infections

In Serbia, the vaccination campaign started in early 2021, only several months after the first described outbreak, and NHs were one of the priority vaccination targets. Thus, all willing residents (94.5%) and 100% staff, naïve or previously infected with SARS-CoV-2, were vaccinated with two doses during February and March. A great majority of the residents received the inactivated whole virus BBIBP-CorV (Sinopharm) vaccine (95%), while only 5% received the BNT162b2 vaccine. The staff was also largely vaccinated with BBIBP-CorV (69.6%), followed by BNT162b2 (17.9%), and two vector vaccines, Gam-COVID-Vac (Sputnik V, Gamaleya) (10.7%) and ChAdOx1 (AstraZeneca) (1.8%). Moreover, the third dose was offered as of August 2021, and was received by 69.1% of all residents at the time (76/110) and 78.6% of all staff (44/56).

**Table 4:** Anti-SARS-CoV-2 specific antibodies in a subgroup of nursing home residents and staff after outbreak 1 in November 2020.

	Residents (n=35)		Staff (n=45)		p (Residents vs. Staff)	
Age [years, mean ± SD (range)]	79.03 ± 7.43 (55-89)		40.11 ± 11.52 (20-59)		<0.0001	
	1^st^ sampling	2^nd^ sampling	1^st^ sampling	2^nd^ sampling	1^st^ sampling	2^nd^ sampling
Average sampling time from diagnosis (days), mean ± SD	40.1 ± 9.4	79.1 ± 6.6	30.4 ± 4.7	68.7 ± 12		
RBD IgG positive (%)	35 (100%) 34 (97.14%)		44 (97.8%) 31 (86.1%)			
RBD IgG [BAU/ml, mean ± SD (range)]	417.3 ± 273.5 (29.68-920.3)	325.7 ± 214.4 (46.35-837.6)	201.9 ± 192.9 (21.35-753.0)	137.7 ± 135.6 (20.33-557.3)	<0.0001	<0.0001
RBD IgM [index, mean ± SD (range)]	7.39 ± 7.65 (1.05-30.01)^*^		3.53 ± 3.68 (1.00-14.40)^**^		0.05	

* n=27;

* **n=23.

**Table 5:** Previous immunization of SARS-CoV-2 infected residents and staff in nursing home.

First two doses						Third dose		
	BBIBP-CorV	BNT162b2	Gam-COVID-Vac	ChAdOx1	BBIBP-CorV	BNT162b2	Gam-COVID-Vac	ChAdOx1
Outbreak 2								
Residents	20/22 (90.9 %)	2/22 (9.1%)	/	/	7/22 (31.8%)	1/22 (4.5%)	/	/
Staff	3/3 (100 %)	/	/	/	2/3 (66.7 %)	/	/	/
Outbreak 3								
Residents^*^	28/36 (77.8 %)	5/36 (13.9%)	/	/	14/36 (38.9 %)	8/36 (22.2 %)	/	/
Staff	14/19 (73.7 %)	3/19 (15.8%)	1/19 (5.3%)	1/19 (5.3%)	8/19 (42.1 %)	4/19 (21.1 %)	1/19 (5.3%)	/

* outbreak three involved 36 residents of whom 3 were not vaccinated

Most notably, all cases in outbreak 2 (22/22 residents and 3/3 staff) and a vast majority of those in outbreak 3 (33/36 and 19/19 staff) were breakthrough infections. Only three patients were not vaccinated, all of whom were residents and all in outbreak 3; interestingly, all these three were primary infections. The distribution of the breakthrough infections according to the vaccine type and number of doses received is presented in Table 5. A great majority of the affected individuals, both residents and staff, in both outbreaks 2 and 3 were vaccinated with BBIBP-CorV (85.5%), while only 12 of the 83 (14.5%) infections in vaccinated individuals occurred after vaccination with any other product. Also, the two deaths that occurred in outbreak 2 were also individuals vaccinated with 2 doses of BBIBP-CorV.

Importantly, a number of patients were involved in more than one outbreak. Four cases of re-infection were registered in outbreak 2 (one resident, three staff) while outbreak 3 even involved 23 reinfected individuals (13 residents, 12 of whom had been infected in outbreak 1 and one in outbreak 2, ten staff, all from outbreak 1). Like with breakthrough infections, cases of reinfection occurred most commonly after vaccination with BBIBP-CorV. Of the total of 27 reinfections, 19 (70.4%) occurred after BBIBP-CorV, six after two and 13 after three doses, and three after two doses of BBIBP-CorV followed by a boost dose of BNT162b2. Four reinfections occurred after three doses of BNT162b2 and one after three doses of Gam-COVID-Vac (Table 6). However, and most importantly, despite the number of reinfections, they were clinically mild, with no severe cases and no lethal outcomes.

**Table 6:** Distribution of breakthrough infections and reinfections in outbreaks 2 and 3 per vaccine type and number of doses received.

		Outbreak 2				Outbreak 3			
		Residents (n=22)		Staff (n=3)		Residents (n=33)		Staff (n=19)	
		2 doses	3^rd^ dose	2 doses	3^rd^ dose	2 doses	3^rd^ dose	2 doses	3^rd^ dose
BBIBP-CorV	All	13*	7	1	2	14	14	6	8
	Reinfections	0	1	1	2	3	7	2	3
BNT162b2	All	2	1**	0	0	1	8 (4 + 4**)	0	4 (3 + 1**)
	Reinfections	0	0	-	-	0	3 (1 + 2**)	-	4 (3 + 1**)
Gam-COVID-Vac	All	0	0	0	0	0	0	0	1
	Reinfections	-	-	-	-	-	-	-	1
ChAdOx1	All	0	0	0	0	0	0	1	0
	Reinfections	-	-	-	-	-	-	-	-

* Two deaths;

* initially received two doses of BBIBP-CorV

## DISCUSSION

Our long-term study of the COVID-19 epidemic in the setting of a single NH allowed us to observe the characteristics of three outbreaks that occurred over a period of two years in one cohort of residents and staff. Multiple outbreaks have been reported in NHs [1] but what is interesting here is that each of the three occurred at a time of the predominance of a different SARS-CoV-2 variant, in both the pre- and post-vaccination periods.

In Serbia, the first case of COVID-19 (Wuhan variant of SARS-CoV-2) was officially diagnosed in the beginning of March 2020, and the first wave was followed by another two waves in June and November of 2020. During the first two waves, infection was avoided by preventive quarantine in NH, but the virus entered the NH during the third wave in November 2020, when forced quarantine was immediately imposed. Therefore, it took the Wuhan variant (or other pre-Alpha variants) 8.5 months to enter the NH. The subsequent variants of SARS-CoV-2 have increased the basic reproduction number (Ro) and effective reproduction number (R_t_) in accordance with their dominant transmission pattern and immunization resistance [17,18]. Vaccination in February 2021 protected residents and staff from the Alpha variant of SARS-CoV-2 during its epidemic wave in the spring of 2021. However, the SARS-CoV-2 Delta variant, which appeared in Serbia in June 2021, entered the NH in the beginning of November 2021. So, it took the Delta variant 4.5 months to enter the NH, a predominantly vaccinated setting at the time. Lastly, the SARS-CoV-2 Omicron variant, registered in Serbia mid-December 2021, entered the NH in the middle of January 2022, which means it took it only one month to enter the NH. These timelines clearly demonstrate the invasiveness and transmission capacity of the SARS-CoV-2 variants in accordance with their novel mutations.

The CFR in the pre-vaccination setting of 31.2% shown in this study is in line with previous studies. An analysis of SARS-CoV-2 infection in 168 Spanish facilities showed 27.7% of residents were infected, and an overall CFR of 24.9% among those with a positive PCR test [19]. Moreover, 68% of UK care homes had at least one COVID-19 infection or related death with CFR rate of 35.7%. Considering residents with no direct evidence of infection, mortality was two-fold higher in care homes with outbreaks versus those without [1]. An investigation of four NHs affected by COVID-19 outbreaks in central London where systemic testing identified 43% of asymptomatic infections in residents, registered 26% of fatalities [2]. NHs offering dementia services had the highest rates of COVID-19 cases (64.0%) and highest proportion of deaths (77.1%) [20]. In our study, dementia and Alzheimer were aggravated by SARS-CoV-2 infection in 17-55% infected residents.

A poorer clinical outcome in unvaccinated residents with COVID-19 in our study was associated with higher leukocyte counts and increased NLRs, as well as with increased CRP and LDH levels. These results repeat published findings; an increased NLR score has been associated with mortality of COVID-19 patients [21,22]; increased levels of CRP (mean 83-mg/l) in patients from NHs at the time of hospital admission have been suggested as a prognostic marker for disease severity and mortality in COVID-19 infection [23], while increased LDH levels have been associated with severe morbidity and mortality in patients with COVID-19 [24]. We found no sex-related differences in inflammation markers in COVID-19 affected residents during the first two outbreaks, but neutrophil counts, NLRs and CRP levels were significantly higher and lymphocytes lower in males in the third outbreak of our study.

Factors related to the risk of infection included larger facility size and higher community incidence, while mortality was associated with male sex, age and higher community incidence [19]. In our study outbreaks only occurred at times of higher community incidence, and mortality was only associated with older adults (as compared to staff members). Male sex has been associated with a higher risk of hospital admission, severe disease and lethal outcome, but showed decreasing associations after age 74 [25-27]. Lower importance of sex with advancing age has been shown in other studies as well [11,12], and may be considered confirmed by our finding of no associations of infection rate, disease severity or lethal outcome with either sex, in a population of a mean age of 81 years.

The dire numbers in COVID-19 affected older adults, the clinical outcome and CFR were all largely altered by vaccination. The proportion of LTCF experiencing outbreaks declined to 4.7% (vs. over 50% in previous period), between May and June 2021 in England, when 64.1% of staff and 85% of residents had been fully vaccinated with two doses [28]. An analysis of 240 COVID-19 outbreaks occurring between July and October 2021 in LTCFs with high vaccination coverage from 10 EU/EEA countries reported 22.2% COVID-19 cases, 17.4% cases of hospitalization, and a CFR of 10.2% [29]. According to our study, immunization (largely with the BBIBP-CorV) prevented poor clinical outcome in the second outbreak and especially in the third outbreak during the waves of the subsequent viral variants, but did not prevent reinfection. This was particularly obvious at the time of the Omicron variant, since a vast majority of all registered infections were breakthrough infections (33/36 in residents, 19/19 in staff). Since this outbreak occurred one year after primo-immunization and five months after the booster dose (received by about two thirds of both the residents and the staff), the number of breakthrough infections is a result of both the high transmission capacity of the Omicron variant and of waning immunity. Low levels of anti-spike IgG antibodies 28 weeks after an mRNA vaccine booster dose in both residents and staff (without a previous SARS-CoV-2 infection), have been associated with a higher risk of SARS-CoV-2 infection and severe COVID-19 [30].

However, very few severe cases and virtually no mortality in our series show the effectiveness of the vaccines in general against severe disease forms and mortality. Nevertheless, it should be pointed out that a great majority of both breakthrough infections and reinfections occurred in individuals vaccinated with BBIBP-CorV, while there were very few such infections after vaccination with any other vaccine.

A most important finding of our study is that it showed a well-preserved immune response in older adults, with higher specific antibody levels detected in the residents than in the NH staff, which corresponded with the clinical severity of COVID-19. This is in contrast to a report showing that SARS-CoV-2 naïve residents had lower antibody responses to BNT162b2 vaccination than naïve staff [31]. A French study in a similar setting showed persistent humoral immunity against SARS-CoV-2 in residents, with a post-vaccination response lasting at least nine months in those who were previously infected, and lower in those who were seronegative before vaccination [32]. This is no different than in other age groups since waning of the vaccinal humoral immune response in the absence of re-stimulation is a normal immunological phenomenon and has been well established after vaccination against SARS-CoV-2 [33-35], leading to introduction of booster doses.

While the strength of our study is an in-depth analysis of multiple outbreaks in one cohort of older adults and staff in the setting of an NH both pre- and post-vaccination, it also has some limitations. These include detection of SARS-CoV-2 antibodies only after the first outbreak thus preventing us to follow the long-term evolution of the humoral immune response, as well as lack of blood parameters for infected staff for comparison with the residents. It will be interesting to monitor this cohort in the future for potential long-term consequences.

In conclusion, SARS-CoV-2 infection induced immune response consistent with clinical presentation in accordance with immunization of residents, through both vaccination and previous infections. Preventive quarantine successfully blocked entry of the virus into the NH, but once the virus entered the NH not even forced quarantine could completely stop infection spreading within the NH. Complete immunization of residents and staff could not prevent infection during the waves of the subsequent more mutated SARS-CoV-2 variants, but vaccines successfully prevented severe COVID-19 presentation and largely reduced mortality.
